# The Efficacy of Interdisciplinary Rehabilitation for Improving Function in People with Chronic Pain

**DOI:** 10.1155/2016/7217684

**Published:** 2016-05-08

**Authors:** Svetlana Kurklinsky, Rachel B. Perez, Elke R. Lacayo, Christopher D. Sletten

**Affiliations:** Department of Pain Medicine, Mayo Clinic Florida, Jacksonville, FL 32224, USA

## Abstract

*Objective.* To examine the efficacy of interdisciplinary rehabilitation for improving function in people with chronic pain.* Design.* Retrospective Chart Review.* Setting.* The Pain Rehabilitation Center (PRC) at a medical center.* Participants.* Individuals admitted to the PRC.* Interventions*. The PRC operates a 3-week outpatient program that utilizes an interdisciplinary approach to treat people with chronic pain. The main treatment elements include physical therapy, occupational therapy, cognitive behavioral therapy (CBT), and medication management. Physical therapy groups focus on moderate exercise despite symptoms. Occupational therapists teach moderation, time management, and activity modification. CBT groups, led by a pain psychologist, address the psychosocial comorbidities of chronic pain. Medical staff oversee the tapering of opiate analgesics and other symptom targeted treatments. This integrated approach is indicated when conventional treatments have been ineffective.* Outcome Measures.* The objective outcome was the 6-minute walk test (6 mWT) distance. The subjective outcomes were performance (COPM-PER) and satisfaction (COPM-SAT) as measured by the Canadian Occupational Performance Measure (COPM).* Results.* Average 6 mWT distances improved by 39% from 375 m to 523 m. Average COPM-PER scores increased from 3.4 to 7.5. Average COPM-SAT scores increased from 2.4 to 7.5.* Conclusions.* Comprehensive interdisciplinary outpatient rehabilitation can significantly improve function in people with chronic pain.

## 1. Introduction

Chronic pain is a complex clinical entity that can affect every aspect of a person's life. The physical consequences of chronic pain include deconditioning, loss of mobility, loss of independence, increased medical comorbidities, and long-term reliance on pain medications. From a psychosocial perspective, these people often experience high levels of emotional distress, insomnia, and impaired social and occupational functioning [1].

Comprehensive, intensive, interdisciplinary, pain rehabilitation programs are safe and effective in treating people with chronic pain [2–5]. A growing body of literature exists to support the immediate and long-term benefits of the interdisciplinary rehabilitation approach [6–9]. In interdisciplinary pain programs the healthcare professionals work from the same facility, with daily meetings about the patients' progress, the same treatment vision is shared, and the same message is passed to the patients.

Multiple studies have reported positive functional outcomes based on patient self-assessment [6–8], but few studies have shown quantitative functional improvement based on objective measures. Previous studies that mostly rely on patient self-report or staff evaluation have demonstrated a reduction in pain symptoms and an improvement in quality of life (QOL) [10, 11]. While the results have been consistent across these studies, there is an inherent limitation to relying on this subjective data. Peppin et al. showed the 6 mWT to be a simple and inexpensive measure of function in the chronic pain population [12].

Our team retrospectively analyzed the objective and subjective functional outcomes of our comprehensive outpatient pain rehabilitation program to assess its efficacy and to guide efforts towards future prospective research.

## 2. Methods

### 2.1. Participants

The authors retrospectively analyzed the 6 mWT and COPM data of the first 150 patients enrolled in the Comprehensive Pain Rehabilitation Center (PRC) program from October 2011 to August 2012. A physician medically cleared each PRC patient to participate in the 3-week rehabilitation program. As part of the regular physical therapy and occupational therapy evaluations and treatment plans, data on the 6 mWT and the COPM were collected and recorded at both the admission and dismissal appointments. Patients voiced an interest in improving daily function and a willingness to taper and cease all pain medications and pain behaviors. Patients with acute pain or illness and those not willing to participate fully in all the components of the treatment program were not admitted to the PRC and thus excluded from this study.

### 2.2. Treatments

The physical therapy component of the interdisciplinary treatment approach focuses on general reconditioning with graded exposure to activity, gradual reduction of fear-avoidance behaviors, and incremental elimination of other pain behaviors. Each patient attends three daily exercise sessions. Morning stretch group is the first session of the day and incorporates 15–20 minutes of whole-body active range of motion, gentle dynamic stretching, moderate static stretching, balance, and coordination training. Patients are encouraged to perform these exercises on the weekend and are instructed in appropriate individual modifications as needed. Cardio and strength groups are also performed, Monday through Friday. Patients are specifically instructed not to perform these exercises on the weekend to minimize maladaptive exercise techniques. During cardio group, each patient's goal is to complete 20–30 minutes of moderate conditioning activities within an age determined target heart rate zone and to cool down from that session with an additional 5–10 minutes of static stretching. During strength group, each patient uses free weights, resistance bands, or body weight resistance to complete a whole-body strengthening and stability circuit. Patients generally finish this session in 45–60 minutes.

The occupational therapy component of the interdisciplinary treatment approach focuses on moderation and balance of daily activity with modification as needed to increase functional independence and participation in life roles. Group lecture topics include bathroom safety, body mechanics, cleaning, cognitive strategies, driving, fall prevention, garage, garbage, home safety, kitchen, laundry, making the bed, moderation, modification, self-care, shopping, time management, values, vocation, workspace ergonomics, and yardwork. Each Friday, the occupational therapists (OTs) lead a time management session for weekend planning to help the patients appropriately moderate their schedule and balance activity during their time away from the structure of the program. The OTs meet with each patient individually on several occasions throughout the program. Midway through the program, on day 8, an OT meets individually with each patient to begin planning his or her days immediately after the program. There are also three individual biofeedback sessions during which patients learn diaphragmatic breathing and muscle relaxation and how to utilize these strategies during daily activity and functional mobility.

A pain psychologist leads up to three group therapy sessions each day. Discussion topics include anger, anxiety, assertiveness, behavior change, central sensitization syndrome, cognitive behavioral therapy, cognitive coping skills, chronic pain cycles, depression, difficult day planning, distraction, drug interventions, fear, forgiveness, goal setting, grief, maintaining lifestyle changes, pain behaviors, PRC programs concepts, perfectionism, personal responsibility, problems solving, relationships, relaxation, self-esteem, sleep, stress, and withdrawal. There are also weekly question and answer sessions for the group members with no scheduled topic and weekly sessions for family and friends to learn about PRC program and ask questions. Each of the discussion sessions is an hour long and group members are encouraged to participate and ask questions as they arise. When there are fewer than three of these lecture sessions in a day, there are work groups for distraction and difficult day planning, nutrition, posture, and PRC program tools that are led by PRC staff and consultants.

Throughout the three-week program, nurses serve as individualized and highly specialized patient care coordinators. They take the lead role in medication management and tapering (under the direction of a physician and physician assistant). With patient authorization, nurses in the program also continually communicate with primary and specialty care providers outside of the PRC team to assist with continuity of care into the future.

### 2.3. Outcome Measures

#### 2.3.1. 6-Minute Walk Test (6 mWT)

The 6 mWT is a submaximal, performance-based measure of functional capacity. It was first defined by Dr. Balke in 1963 [2] and has since been used in a variety of populations as a reliable [13, 14], valid [15–17], sensitive [18], and specific [19] measure of functional capacity. It has a good predictive value of morbidity and mortality [20, 21], as well as disability status [22]. Normative data has been collected [23] on the healthy adult population in order to establish statistically and clinically meaningful changes [24–26]. An improvement of 54 m is significant for a change in functional status [26]. Walking speed is a function of distance and time and can be derived from the 6 mWT. Speeds of less than 1.0 m/s have been associated with physical and cognitive decline and decreased independence with activities of daily living [27, 28].

For each patient, one of the four physical therapists (PTs) performed the 6 mWT. A 30.48 m (100 foot), demarcated, up-and-back walkway was used along with a stopwatch to keep time. Instructions given before the test began were to walk up and down the hallway safely, covering as much distance as possible in 6 minutes, without running. Patients were further instructed to only take rest breaks if needed, but the time would continue to count down. During the test, time updates were provided every 30–60 seconds without encouragement.

### 2.4. The Canadian Occupational Performance Measure (COPM)

The COPM is a quality-of-life measure designed to capture limitations in relation to activities of daily living. The patients rate their performance and satisfaction in the areas of self-care, productivity, and leisure. The COPM has been validated in diverse patient populations [29] including stroke [30, 31], pain [32], and cerebral palsy [33]. The COPM was defined in 1991 and has since been used in more than 35 countries. In Canada, it represents the national standard for clinical practice and research in occupational therapy [34]. The materials required to perform the COPM are a test form and scoring cards.

The COPM was administered in a semistructured interview format by 1 of 2 OTs during the occupational therapy evaluation. The patient was asked to provide a self-report of occupational performance limitations and to rate each problem on level of importance (1–10, 1 = not important at all; 10 = extremely important). The patient was then asked to rate their performance for the top five most important items (1–10, 1 = not able to do it; 10 = able to do it extremely well). Lastly, they were asked to rate their current satisfaction (1 = not satisfied at all; 10 = extremely satisfied) with that performance level.

Note that the OT did not reveal to the patient their initial scoring until after the discharge scoring was complete to help reduce any scoring bias.

#### 2.4.1. Statistical Analysis

All statistical analyses were performed using JMP computer program (Cary, NC), version 10.0.0. The results were considered statistically significant at the 0.05 level. Confidence level was set at 95%. The data were analyzed using ANOVA test assuming equal variance. This was done to minimize error variance and more accurately detect pre- to posttreatment differences.

## 3. Results

These PRC patients had a primary diagnosis of central sensitization syndrome with mixed pain sources reported (30% fibromyalgia, 20% low back pain, 11% abdominal pain, 4% headache, and 35% mixed/others). Out of the 150 patient charts that were retrospectively analyzed, 132 patients successfully finished the program, 17 patients quit before finishing the program, and one patient's 6 mWT data was not documented. The 17 patients that left the program early had similar demographics and pain conditions as the rest of the patients. The gender distribution of patients was 114 females (76%) and 36 males (24%). The average age was 51 years, with a range from 19 to 85 years. Demographics of admitted patients were as follows: 89% white, 5% black, and 1% Asian, and the remaining 5% declared themselves as others (Table 1). The education level of admitted patients was variable: 30% completed some college/associates degree, 27% graduated from college, 22% have postgraduate education, 19% are high school graduate/GED, 2% did not graduate from high school. Average pain duration for admitted patients was 10.9 years (Table 1). The average numeric pain score reported at the admission was 6.4 and 4.9 at the dismissal (*p* < 0.0001). Over three-quarters (78%) of the admitted patients were employed before the initial onset of chronic pain, yet only 21% of them worked at the time of admission.

**Figure 1:**
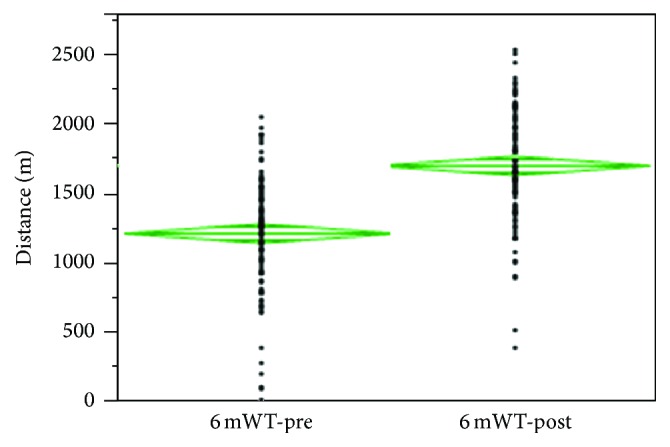
Distribution of the 6-minute walk test at the beginning (6 mWT-pre) and at the end (6 mWT-post) of the PRC program. ANOVA test assuming equal variance; the mean difference was 484.65 m, the standard error difference was 49.36, and Prob < 0.0001. Confidence level was 95%. All but 2 patients improved their 6 mWT by the end of the PRC program.

At admission, patients walked an average of 375 m (3750 m/h, 1.0 m/s) during the 6 mWT (Figure 1). The same patients walked an average of 523 m (5227 m/h, 1.4 m/s) at dismissal (Figure 1), which is a 148 m increase and a 39% improvement in functional capacity. The means between 6 mWT-pre and 6 mWT-post are statistically different by *t*-test analysis, with *p* value less than 0.0001.

**Figure 2:**
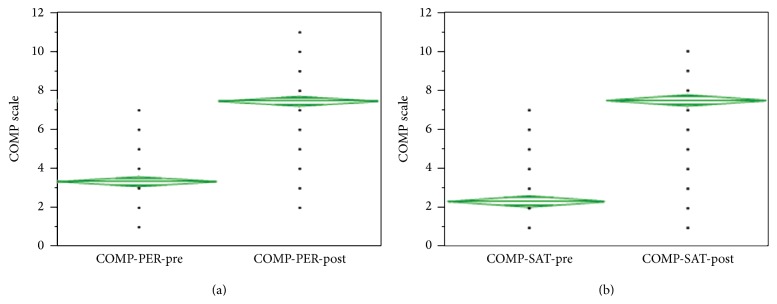
(a) Distribution of the COPM performance scores at the beginning (COMP-PER-pre) and at the end (COMP-PER-post) of the program. ANOVA assuming equal variance; the difference of the means was 4.12, standard error difference was 0.18, and Prob < 0.0001. Confidence level was 95%. All but 1 patient improved COMP performance scores. (b) Distribution of the COPM satisfaction scores at the beginning (COMP-SAT-pre) and at the end (COMP-SAT-post) of the program. ANOVA assuming equal variance; the difference of the means was 5.11, standard error difference was 0.206, and Prob < 0.0001. Confidence level was 95%. All but 1 patient improved COMP satisfaction scores.

At admission, patients rated themselves an average of 3.4 and 2.4 out of 10 for the COPM performance (COPM-PER-pre) and satisfaction (COMP-SAT-pre) scores, respectively (Figure 2(a)). At dismissal, the same patients scored an average of 7.5 in both categories (COMP-PER-post and COMP-SAT-post) (Figure 2(b)). The probability value for mean differences of COPM performance and satisfaction scores was less than 0.001 using Wilcoxon's test analysis.

## 4. Conclusions

In many cases of chronic pain, significant pain reduction is not possible, whereas increasing function and quality of life is a reasonable and achievable goal. Interdisciplinary pain rehabilitation programs have been successful in the rehabilitation of people with chronic pain. We use the 6 mWT and COPM to assess functional capacity and occupational performance in all of our patients at admission and dismissal. We retrospectively assessed the progress and satisfaction of the first 150 patients enrolled in our PRC program.

The distance covered in the 6 mWT correlates with functional capacity and independence with activities of daily living. Walking speed has been referred to as the sixth vital sign [35]. Poor walking tolerance is linked to an increased likelihood of adverse events such as falls, hospitalization, and overall disability [22, 36–38]. Patients finishing our PRC program on average saw a 39% improvement (148 m) in their 6 mWT distance. This improvement is nearly 3 times the substantially meaningful change (50 m) previously established by Perera et al. [25].

Changes in COPM scores from assessment to reassessment tend to be meaningful [39] and a change of 2 or more points on any single item has been shown to be clinically significant [34]. Patients finishing our PRC program on average reported increases of 4.1 points and 5.1 points, respectively, for the means of COPM performance and satisfaction scores. This clearly demonstrates clinical significance and parallels the objective findings of the 6 mWT.

These findings add to the well-established pain rehabilitation literature. The PRC program positively impacts functioning for a broad range of people. By using the 6 mWT, we have objectively demonstrated that improvements are clinically meaningful, as patients' progress from needing interventions to reduce fall risk to being independent in activities of daily livings.

The main limitation of this retrospective study design is the lack of an appropriate control group; however, each patient's pre-PRC program data is a representation of their own status having tried many of the conventional treatment options. Future studies would be beneficial to analyze these effects prospectively and a longitudinal follow-up study would be able to investigate if these positive results persist over time.

## Figures and Tables

**Table 1:** Patients' demographics at the time of admission to the PRC program.

Baseline characteristics	*n*
Gender	
Male	36
Female	114

Age (avg. yr)	51

Education (yr)	
<12	4
12	28
13–15	44
>15	74

Race	
White	134
Black	7
Asian	2
Others	7

Average pain duration (yr)	10.9

Marital status	
Married	103
Single	23
Separated	4
Divorced	17
Widowed	3
